# Network analysis of smartphone addiction and sleep disorder symptoms in Chinese college students

**DOI:** 10.1371/journal.pone.0349016

**Published:** 2026-05-22

**Authors:** Xiaonan Li, Lin Mao

**Affiliations:** 1 Department of Sociology, Guizhou Minzu University, Guiyang, Guizhou, China; 2 Department of Management, North Sichuan Medical College, Nanchong, Sichuan, China; University of Nis Faculty of Medicine: Univerzitet u Nisu Medicinski Fakultet, SERBIA

## Abstract

**Objective:**

This study aims to examine the comorbid relationship between smartphone addiction and sleep disorders in Chinese college students. By constructing a comorbidity network, identifying core and bridge symptoms, and exploring potential directional associations among symptoms, this research intends to establish a theoretical foundation for targeted intervention strategies.

**Methods:**

A total of 1842 Chinese college students were recruited through convenience sampling. The smartphone addiction and sleep disorder symptoms were assessed using the Smartphone Addiction Scale-Short Version (SAS-SV) and the Pittsburgh Sleep Quality Index (PSQI), respectively. The data analysis was conducted in three steps. First, an undirected comorbidity network was constructed using the Gaussian Graphical Model (GGM) to identify core and bridge symptoms. Second, a Bayesian network approach was employed to generate Directed Acyclic Graphs (DAGs) that explored potential directional associations among symptoms. Finally, network comparison tests and community detection analyses were performed to examine gender differences in the comorbidity network structure.

**Results:**

The GGM comorbidity network exhibited a connection density of 0.80 and a global strength of 9.39. Within this network, PSQI2 (sleep latency), SA2 (difficulty concentrating), and SA5 (impatience without phone) were identified as core symptoms. PSQI2 (sleep latency), PSQI1 (subjective sleep quality), and SA9 (longer use than intended) were identified as bridge symptoms. Further analysis using the DAGs suggested statistical directionality from sleep disorder symptoms toward smartphone addiction symptoms. Notably, SA5 (impatience without phone) served as an initial node in the DAGs. Finally, network comparison tests indicated no significant differences in the GGM network structure between genders; however, distinct gender differences were observed in the community clustering patterns of symptoms.

**Conclusion:**

In college students, smartphone addiction and sleep disorder symptoms interact to form a structurally stable comorbidity network. Consequently, interventions targeting core symptoms, bridge symptoms, and initial node could effectively interrupt the maintenance of this comorbidity.

## Introduction

Driven by the rapid development of information technology and the widespread adoption of the Internet, smartphones have achieved unprecedented popularity. According to the Statistical Report on Internet Development in China, as of June 2025, there were approximately 1.123 billion internet users in China, reflecting a penetration rate of 79.7%. Notably, 1.116 billion of these users accessed the internet via mobile devices, accounting for 99.4% of the total online population [1]. While smartphones offer considerable convenience, they have also engendered concerning issues such as overuse and addiction [2]. Smartphone addiction is defined by excessive reliance on or compulsive use of smartphones, which can result in a range of psychological and behavioral problems [3]. Smartphone addiction impairs academic performance [4], damages interpersonal relationships [5], reduces life satisfaction [6], and negatively impacts mental health [7]. Moreover, it can impair cognitive function [8] and physical health [9]. Given that college students are in a critical stage of psychosocial development with immature self-regulation skills [10] and considerable discretionary time [11], they are particularly susceptible to smartphone addiction [12]. Against this backdrop, conducting a network analysis of smartphone addiction symptoms among college students is of considerable theoretical and practical relevance.

Sleep disorders encompass a range of conditions that disrupt normal sleep patterns, typically manifesting as difficulty falling asleep, early awakening, and excessive daytime sleepiness [13,14]. These symptoms can not only significantly diminish an individual’s sleep quality but also impair their daytime functioning. Among college students, the prevalence of sleep disorders is notably high. A meta-analysis reported that approximately 25.7% of Chinese college students experience sleep disorders, including 20.3% who report poor sleep quality and 23.6% who exhibit insomnia symptoms [15]. Moreover, during the normalization phase of COVID-19 prevention and control in China, a survey of 1326 college students found that 32.20% of respondents reported poor sleep quality [16]. Poor sleep quality significantly affects college students’ academic performance, emotional well-being, and physical health [17,18], warranting urgent attention and intervention.

There is a bidirectional relationship between smartphone addiction and sleep disorders [19, 20]. On the one hand, smartphone addiction is a significant risk factor for sleep disorders. Multiple studies have demonstrated that smartphone addiction is positively associated with sleep disorders, and the risk of sleep problems among addicts is significantly higher than that among non-addicts [21, 22]. The underlying mechanism is reflected in the following aspects. First, the electromagnetic field emitted by smartphones may increase electroencephalogram spectral power and reduce slow-wave sleep duration, thereby impairing sleep quality [23]. Second, long-term use of mobile devices may induce physical discomfort such as muscle pain and headaches, thereby increasing the risk of sleep disorders [24,25].Third, using a mobile phone near bedtime can disrupt the body’s circadian rhythm, delay the initiation of sleep signals, and thereby disturb normal sleep patterns [26]. On the other hand, sleep disorders may also induce or exacerbate smartphone addiction [27]. The Interaction of Person-Affect-Cognition-Execution (I-PACE) model proposes that when an individual experiences insomnia or sleep deprivation, their emotional regulation and executive functions are likely to become impaired. Consequently, they become more predisposed to seek immediate comfort and escape from reality via smartphones [28]. Similarly, the Compensatory Internet Use Theory (CIUT) posits that excessive internet use serves as a coping strategy to alleviate negative emotions [29]. As demonstrated by Tavernier and Willoughby [30], individuals with sleep difficulties exhibit a higher frequency of social media and other mobile application use. Such negative coping mechanisms can potentially trap individuals in a self-reinforcing cycle: sleep disturbances prompt compensatory smartphone use, which in turn further aggravates sleep problems.

While existing studies have established an association between smartphone addiction and sleep disorders, traditional research has primarily analyzed them as latent variables composed of symptom clusters. This approach can explain the overall associations between latent variables but often fails to elucidate the interaction mechanisms among specific symptoms [31,32]. According to the network theory of mental disorders, one symptom can directly influence another, leading to mutually influential relationships [33]. Therefore, understanding the interactions between symptoms is crucial for comprehending the occurrence and development of psychological disorders, including smartphone addiction and sleep disorders. Network analysis, a statistical method widely used in psychology, can quantify the relationships between individual symptoms [34]. This method typically uses the Gaussian Graphical Model (GGM) to estimate partial correlation networks of mental disorders, where symptoms are treated as “nodes” and the partial correlation coefficients between symptoms are treated as “edges.” However, a significant limitation of partial correlation networks is that they typically cannot infer directional relationships between symptoms [35]. In contrast, Bayesian networks provide a more advanced network estimation method that can infer potential directional associations from cross-sectional data [36]. This approach not only helps to explore statistical directionality between symptoms but also identifies key symptoms that may have a significant impact without relying on centrality indices.

Therefore, this study aims to systematically investigate the comorbid relationship between smartphone addiction and sleep disorders among college students using network analysis. First, we will employ a Gaussian Graphical Model (GGM) to construct an undirected comorbidity network of smartphone addiction and sleep disorder symptoms. This model allows us to calculate network centrality metrics to identify core and bridge symptoms, thereby clarifying symptom correlation patterns and comorbidity network structure. Next, we will apply Bayesian network analysis to infer directed acyclic graphs from cross-sectional data, which can elucidate potential statistical directionality among symptoms. Finally, we will estimate GGM networks for male and female college students to conduct a comparative analysis. By visualizing the network structures and performing statistical comparisons, we will explore gender differences in the comorbidity network, which can provide an empirical basis for developing targeted prevention and intervention strategies for different gender groups.

## Methodology

### Data and samples

This study was conducted from June to November 2025. Data were collected offline via paper-based questionnaires using a convenience sampling method. Compared to online surveys, paper-based questionnaires tend to yield higher response rates and less careless responding, as researchers can provide on-site supervision to ensure that participants complete the questionnaire attentively. The specific sampling process involved two approaches. First, researchers recruited college students on-site in public areas such as classrooms, libraries, and canteens at two universities in Guiyang and Nanchong. Second, as university staff members, the researchers contacted some class leaders, who assisted in distributing and collecting questionnaires within their respective classes. The inclusion criteria for participants were (1) currently enrolled university students; (2) aged ≥ 18 years; (3) understanding the study content and procedures and voluntarily participating in the survey; and (4) capable of independently understanding and completing the questionnaire. Questionnaires were excluded if they had (1) missing responses to key items in the questionnaire, or (2) obvious logical inconsistencies in the responses. A total of 1980 questionnaires were collected. After excluding 138 invalid questionnaires due to missing key information or logical inconsistencies, 1842 valid questionnaires were ultimately retained, resulting in a valid response rate of 93.03%. The demographic characteristics of the sample are detailed in Table 1. The average age of all participants was 21.03 years (SD = 1.92). In terms of gender distribution, there were 968 females (52.55%) and 874 males (47.45%). Regarding grade composition, the sample included 334 freshmen (18.13%), 436 sophomores (23.67%), 463 juniors (25.13%), 370 seniors (20.09%), and 239 students from other grades (12.98%). Concerning major distribution, the largest proportions were students majoring in Humanities and Social Sciences (30.45%) and Medical Sciences (29.26%).

**Table 1:** Sociodemographic Characteristics of The Sample. (N = 1842).

Sociodemographic characteristics	*N*/ Mean	Percentage(%)/ (± SD)
**Gender**		
Female	968	52.55%
Male	874	47.45%
**Age**	21.03	1.92
**Grade**		
Freshman	334	18.13%
Sophomore	436	23.67%
Junior	463	25.13%
Senior	370	20.09%
Other	239	12.98%
**Major**		
Science and Engineering	95	5.16%
Economics and Management	293	15.91%
Humanities and Social Sciences	561	30.45%
Medical Sciences	539	29.26%
Arts	273	14.82%
Other	81	4.40%

This study was approved by the Ethics Review Committee of the School of Sociology at Guizhou Minzu University. After fully understanding the purpose, content, and procedures of the study, all participants voluntarily signed written informed consent forms. The survey process strictly complied with academic ethical standards. Participants’ personal information is kept strictly confidential, and the collected data are used solely for academic research purposes.

### Measurement

#### Smartphone addiction scale.

This study used the “Smartphone Addiction Scale-Short Version” (SAS-SV), developed by Kwon et al., to assess the level of smartphone addiction among college students [37]. Although originally designed for adolescents, the scale has been widely adopted in related research due to its brevity, reliability, and validity, and its applicability has also been demonstrated in Chinese college student populations [38–40]. The SAS-SV is a self-report questionnaire consisting of 10 items rated on a 6-point Likert scale (1 = “strongly disagree,” 6 = “strongly agree”). Thus, total scores on the scale range from 10 to 60, with higher scores indicating more severe smartphone addiction. In the present sample, the scale showed good internal consistency, with a Cronbach’s α of 0.846.

#### Pittsburgh sleep quality index.

The Pittsburgh Sleep Quality Index (PSQI) was used to evaluate the sleep quality of participants over the preceding month. Developed by Buysse et al., the PSQI includes 19 self-rated items that are grouped into seven components: subjective sleep quality, sleep latency, sleep duration, habitual sleep efficiency, sleep disturbances, use of sleeping medication, and daytime dysfunction [41]. PSQI has been validated as an effective tool for assessing sleep disorders among Chinese university students [42–44]. Each component is scored from 0 to 3. These component scores are summed to produce a global PSQI score ranging from 0 to 21, with higher scores indicating poorer sleep quality. The scale has shown good reliability and validity in Chinese college student populations [45,46]. In the present study, the PSQI demonstrated good internal consistency, with a Cronbach’s α coefficient of 0.771.

### Statistical analysis

#### Estimation and visualization of GGM network.

The study employed the Gaussian Graphical Model (GGM) to construct an undirected network illustrating the relationships between symptoms of smartphone addiction and sleep disorders. Network estimation and visualization were conducted using the qgraph and bootnet packages in R [47, 48]. Specifically, network estimation was performed with the estimateNetwork function using the EBICglasso algorithm. This approach performs regularized model selection by combining the extended Bayesian information criterion (EBIC) with the least absolute shrinkage and selection operator (LASSO). By selecting a penalty parameter, the method regularizes the covariance matrix of symptoms, thereby reducing potential spurious correlations to zero and ultimately estimating a sparse network structure [47]. This study set the gamma parameter in EBIC to the default value of 0.5 recommended in relevant literature [47]. Subsequently, node predictability for each symptom was calculated using the mgm package. This index ranges from 0 to 1, quantifying how much a node’s variance is explained by its neighboring nodes [49]. A higher value indicates greater influence on other symptoms in the network. Finally, in the network visualization, nodes represent symptoms, and edges correspond to partial correlations between nodes. Thicker edges indicate stronger associations, and shorter distances between nodes reflect stronger connection strength.

#### Centrality estimation.

Node centrality measures the relative importance of a node within a network. In this study, the influence of each node in the undirected network was assessed using Expected Influence (EI). The EI value for a node is calculated as the sum of the partial correlation coefficients between that node and all its directly connected nodes. During the calculation process, negative edge weights were retained in their original signed form and were not converted to absolute values [50]. Nodes with high centrality are considered core symptoms, indicating that they have stronger statistical connectivity in the symptom network and show closer associations with other symptoms [31, 51]. Furthermore, following the recommendation of Jones et al., this study utilized the bridge expected influence index (BEI) to identify bridge nodes connecting smartphone addiction and sleep disorder symptoms [52]. When calculating the BEI of a node, only edges connecting it to nodes in other subnetworks are considered, excluding any connections within its own subnetwork. Consequently, a higher BEI value signifies that the node is more strongly linked to symptoms in the other subnetworks.

#### Accuracy and stability test.

In network analysis, “accuracy” refers to the proximity of estimated values to the true parameter values. To assess the accuracy of the GGM network, the non-parametric bootstrap approach (n = 1500) was employed to compute 95% confidence intervals (CIs) for edge weights. The width of CIs reflects the estimation accuracy of edge weights, with wider intervals indicating lower precision [47]. “Stability” indicates the consistency and robustness of network analysis results. First, the case-dropping subset bootstrap approach (n = 1500) was implemented to evaluate the stability of each node’s centrality. Specifically, it assessed whether the centrality-index ranking changed when the network was re-estimated using a smaller sample size. Second, the centrality stability coefficient (CS-coefficient) was calculated using the corStability function in the bootnet package. A CS value above 0.25 is considered acceptable, and above 0.50 indicates good stability [47]. Finally, a bootstrapped difference test (α = 0.05) was conducted on the differences between edge weights and centrality indices across nodes, thereby providing additional evidence of the GGM network stability.

#### Estimation and visualisation of Bayesian network.

To address the limitation that undirected networks cannot reveal the statistical directionality between nodes, this study constructed directed acyclic graphs (DAGs) to investigate the potential directional associations between smartphone addiction and sleep disorder symptoms. The DAG was estimated using the Bayesian hill-climbing algorithm, which iteratively adds, deletes, or reverses edges to optimize the Bayesian Information Criterion (BIC) score, thereby determining the optimal network structure [53]. To ensure the structural stability of the DAG, we performed 2000 bootstrap resamplings and estimated the network for each bootstrap sample separately. First, we used the criteria proposed by Scutari and Nagarajan [54] to determine the optimal significance threshold and then filtered the edges based on their occurrence frequency across 2000 bootstrap resamples, thereby constructing a network with high sensitivity and specificity. Second, we determined the direction of directed edges based on their frequency of appearance in the bootstrap networks: if a directed edge appeared in at least 51% of the bootstrap networks, its corresponding direction was retained in the final DAG. In DAG, the thickness of the edges represents their relative BIC values or probabilities; thicker edges represent more important connections in model fitting and would cause potential detriment to the model fitting effect if removed. The vertical positioning of the nodes reflects their relative importance. The higher the position, the stronger the influence of the node in the network [53].

#### Network comparison.

This study conducted a Network Comparison Test (NCT) with 1000 permutation tests to compare undirected networks between male and female groups [55]. Existing literature indicates that the relationship between smartphone addiction and sleep disorders among college students differs by gender [56], which may lead to variations in symptom interactions across different gender groups. Therefore, analyzing differences in network structures between genders has important practical implications for developing personalized clinical intervention programs. Specifically, we assessed the global and local invariance of the GGM networks. The global invariance test includes two components: the network invariance test and the global strength invariance test. The test statistic for network invariance is the maximum absolute difference in edge weights between the two networks. The test statistic for global strength invariance is the difference in the sum of absolute edge weights across both networks. Local invariance concerns the differences in edge weights and node centrality indices between the two sample networks. Additionally, this study also compared differences in network community structure between male and female samples using a community detection algorithm. This modularity-based algorithm identifies subnetworks by assuming that connections within a given community are significantly stronger than connections to nodes outside of it [57].

## Results

### Descriptive statistics

**Fig 1:**
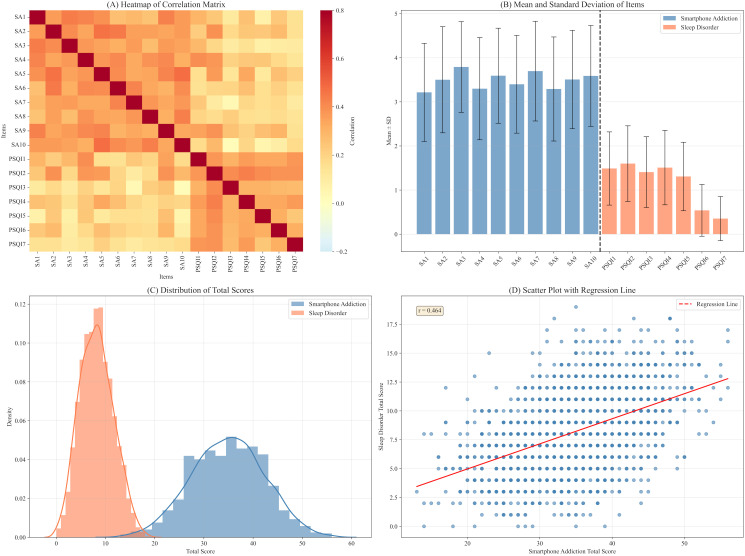
Associations and Distributions of Smartphone Addiction (SA) and Sleep Disorders (PSQI). **(A)** Correlation heatmap of SA1–SA10 and PSQI1–PSQI7. **(B)** Means and standard deviations of each item, with error bars representing the standard deviation. **(C)** Density and histogram of the total scores for smartphone addiction and sleep disorders. **(D)** Scatter plot with a fitted regression line showing the relationship between total SA and PSQI scores; the Pearson correlation coefficient (r) is displayed in the upper left corner.

This study employed the Smartphone Addiction Scale (SA) and the Pittsburgh Sleep Quality Index (PSQI) to assess the smartphone usage behaviors and sleep disorders of participants. The specific meanings and scores for each item are detailed in Table 2. The mean total score on the SA was 34.89 (SD = 7.38). The three items with the highest scores were SA3 (M = 3.79, SD = 1.03), SA7 (M = 3.70, SD = 1.13), and SA2 (M = 3.50, SD = 1.20). For the PSQI, the mean total score was 8.20 (SD = 3.45), with the two components that obtained the highest scores being PSQI2 (M = 1.60, SD = 0.86) and PSQI1 (M = 1.49, SD = 0.83). To further investigate the association between smartphone addiction and sleep disorders, this study analyzed the correlations and distribution characteristics among various symptoms and explored the overall association patterns among variables. The correlation matrix heatmap presented in Fig 1A shows that the majority of relationships between smartphone addiction and sleep disorder symptoms are positive. In this figure, darker colors signify stronger correlations between the symptoms. This matrix is not derived from network analysis, but rather serves to preliminarily assess the strength of correlations between symptom items, thereby laying the foundation for the feasibility of subsequent network estimation. Fig 1B displays the means and standard deviations for each item, facilitating an intuitive comparison of the central tendency and dispersion among symptoms. The histograms and density curves in Fig 1C indicate that total scores of smartphone addiction and sleep disorders approximately follow a normal distribution. Specifically, the density curve of smartphone addiction scores is flatter, indicating a more dispersed data distribution, while the density curve of sleep disorder scores is steeper, suggesting a more concentrated data distribution. Fig 1D shows a scatter plot with a fitted regression line, revealing a linear trend between total scores for smartphone addiction and sleep disorders. The correlation coefficient (r = 0.464) indicates that higher levels of smartphone addiction are associated with more severe sleep disorders. In summary, these findings provide preliminary empirical evidence for the subsequent exploration of the relationship between smartphone addiction and sleep disorder symptoms.

**Table 2:** Descriptive Statistics and Predictability of Items.

Abbreviations	Item	Mean(SD)	Predictability
**SA**	Smartphone addiction scale	34.89(7.38)	–
**SA1**	1.Missing planned work due to smartphone use	3.22(1.11)	0.376
**SA2**	2.Having a hard time concentrating in class, while doing assignments, or while working due to smartphone use	3.50(1.20)	0.419
**SA3**	3.Feeling pain in the wrists or at the back of the neck while using a smartphone	3.79(1.03)	0.344
**SA4**	4.Won’t be able to stand not having a smartphone	3.30(1.16)	0.442
**SA5**	5.Feeling impatient and fretful when I am not holding my smartphone	3.59(1.08)	0.445
**SA6**	6.Having my smartphone in my mind even when I am not using it	3.40(1.11)	0.400
**SA7**	7.I will never give up using my smartphone even when my daily life is already greatly affected by it	3.70(1.13)	0.338
**SA8**	8.Constantly checking my smartphone so as not to miss conversations between other people on Twitter or Facebook	3.29(1.18)	0.344
**SA9**	9.Using my smartphone longer than I had intended	3.51(1.12)	0.400
**SA10**	10.The people around me tell me that I use my smartphone too much	3.59(1.14)	0.425
**PSQI**	Pittsburgh sleep quality index	8.20(3.45)	–
**PSQI1**	1.Subjective sleep quality	1.49(0.83)	0.396
**PSQI2**	2.Sleep latency	1.60(0.86)	0.504
**PSQI3**	3.Sleep duration	1.41(0.80)	0.288
**PSQI4**	4.Habitual sleep efficiency	1.51(0.84)	0.347
**PSQI5**	5.Sleep disturbances	1.31(0.78)	0.280
**PSQI6**	6.Use of sleeping medication	0.54(0.59)	0.297
**PSQI7**	7.Daytime dysfunction	0.35(0.50)	0.282

### Results of GGM network analysis

**Fig 2:**
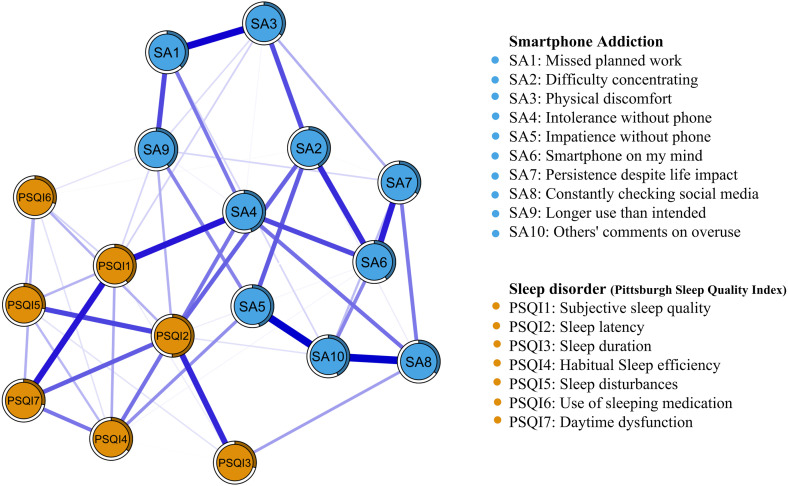
Gaussian Graphical Model (GGM) Network of Smartphone Addiction and Sleep Disorder Symptoms. Nodes represent symptoms; edges represent regularized partial correlations.

**Fig 3:**
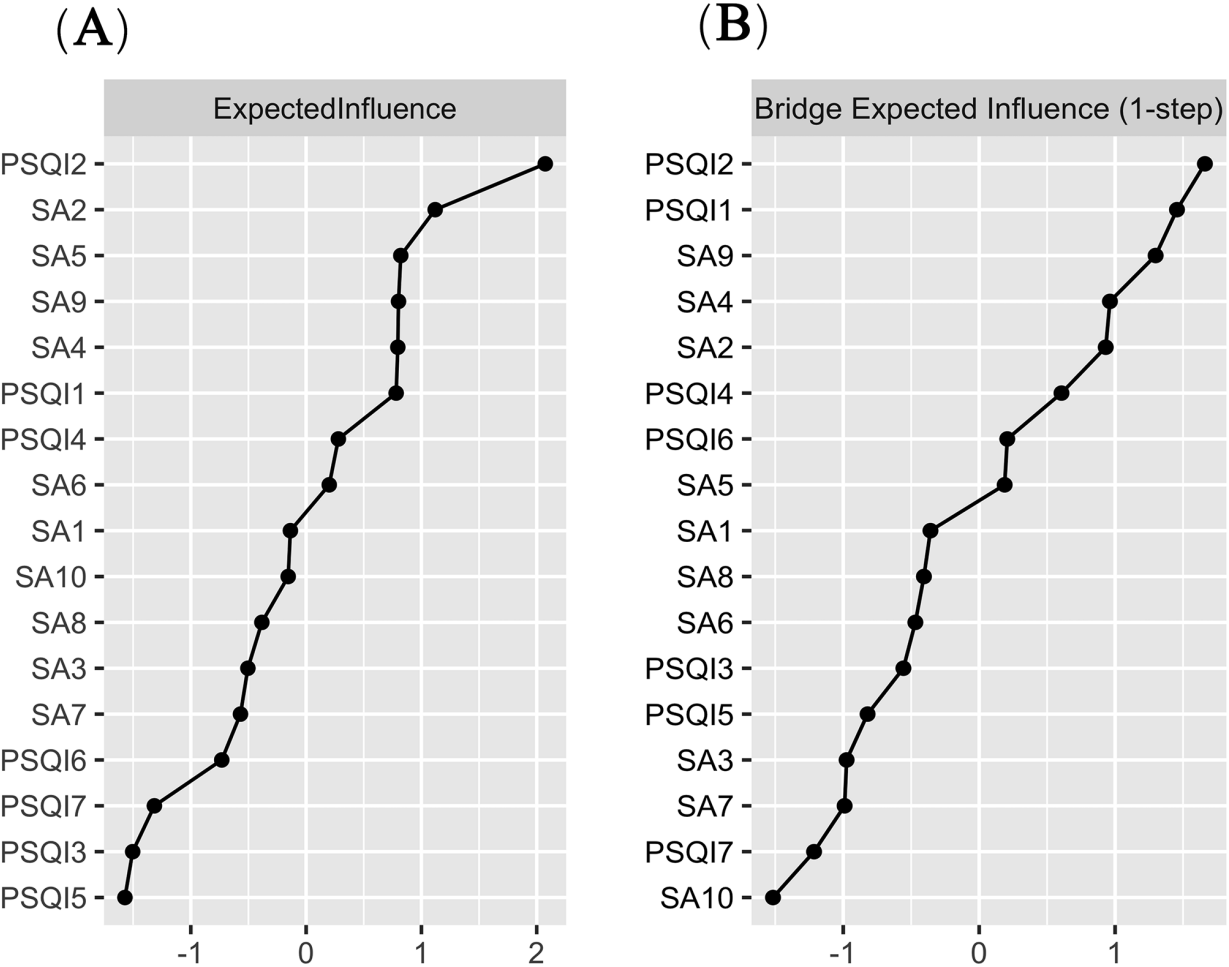
Expected Influence and Bridge Expected Influence of Symptoms. **(A)** The Expected Influence for each symptom in the Gaussian Graphical Model network. **(B)** The Bridge Expected Influence (BEI, 1-step) for each symptom in the Gaussian Graphical Model network.

To examine the relationships between smartphone addiction and sleep disorder symptoms, we estimated a Gaussian Graphical Model (GGM) network that is illustrated in Fig 2. We evaluated the overall connectivity of the GGM network using two key indicators: network density and global strength. Network density is defined as the ratio of the number of actual edge connections to the total number of possible edge connections. Global strength is the sum of the absolute values of all edge weights within the network. The model identified 109 actual edges out of 136 possible edges, yielding a network density of 0.80. Such a high density may raise concerns about insufficient regularization. To address this, we reported the EBIC gamma parameter used in the regularization process. The GGM was estimated using the EBICglasso method with the default parameter γ = 0.5. When γ was set to 0.25 and 0.75, the network densities were 0.86 and 0.79, respectively. These results indicate that even when the regularization strength is adjusted, the network density remains high, and therefore the high density is not due to insufficient regularization. Instead, the high density is more likely attributable to the strong correlations among items measuring smartphone addiction and sleep disorders in our sample (N = 1842), which is consistent with the results of the correlation matrix heatmap presented in Fig 1A. The global strength of the GGM network was 9.39, with an average edge weight of 0.09. These findings suggest that the GGM network exhibits high overall connectivity, implying generally close relationships among the symptoms. The predictability of all nodes is presented in Table 2. Figs 3A and 3B present the Expected Influence (EI) and Bridge Expected Influence (BEI) for each symptom in the GGM network. PSQI2, SA2, and SA5 exhibited the highest EI values, indicating that these symptoms exert the strongest direct influence on other symptoms within the network. PSQI2, PSQI1, and SA9 exhibited the highest BEI values, suggesting their critical bridge role connecting smartphone addiction and sleep disorder symptoms. Notably, PSQI2 ranked highest in both EI and BEI, indicating it is not only a core symptom in the comorbidity network but also a critical bridge node linking the two symptom clusters.

### Accuracy and stability of the GGM network

**Fig 4:**
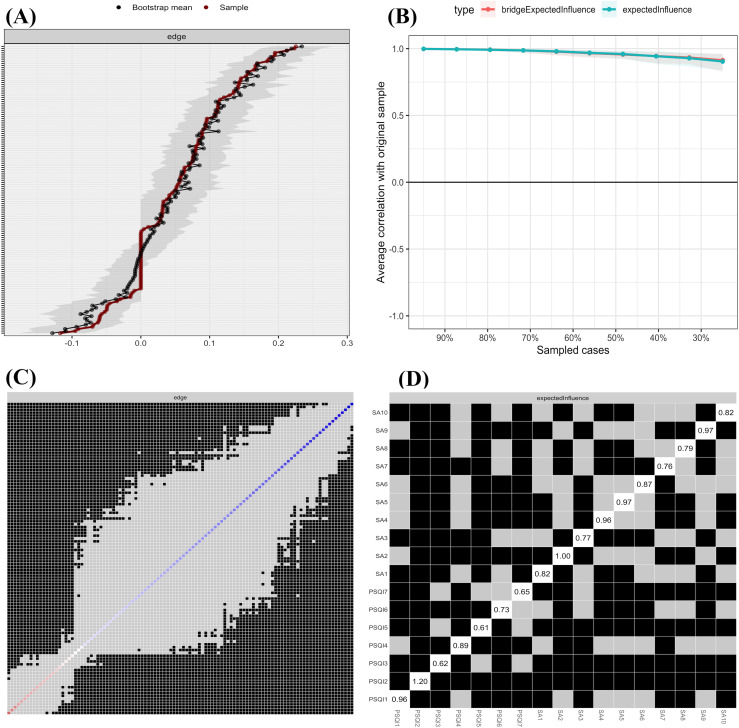
Accuracy and Stability Evaluations of the Gaussian Graphical Model (GGM) Network. **(A)** Bootstrap confidence intervals for edge weights. **(B)** Case-dropping bootstrap stability assessment of centrality indices. **(C)** Significance test for differences in edge weights: black grids indicate significant differences (p < .05), gray grids indicate non-significant differences. **(D)** Significance test for differences in node centrality.

As shown in Fig 4A, the CIs of most edge weights are very narrow, reflecting the high accuracy of edge estimation. For stability assessment, the case-dropping bootstrap results shown in Fig 4B indicate that node centrality rankings remained highly consistent even with reduced sample sizes. The specific results of the stability analysis for the centrality indices are presented in S1 Table. Specifically, the correlation stability (CS) coefficients for Expected Influence and Bridge Expected Influence were both 0.75. This value is well above the recommended threshold of 0.50 for good stability, further confirming the robustness of these centrality measures. Furthermore, Fig 4C and Fig 4D present the results of the difference tests for edge weights and node centralities. In these figures, black grids signify significant differences between node centralities or edge weights, while gray grids denote non-significant differences. The greater the number of black grids, the better the discrimination and structural stability of the network. In our results, most node centralities and edge weights differed significantly, indicating that the GGM network possesses good structural stability.

### Results of DAG network analysis

**Fig 5:**
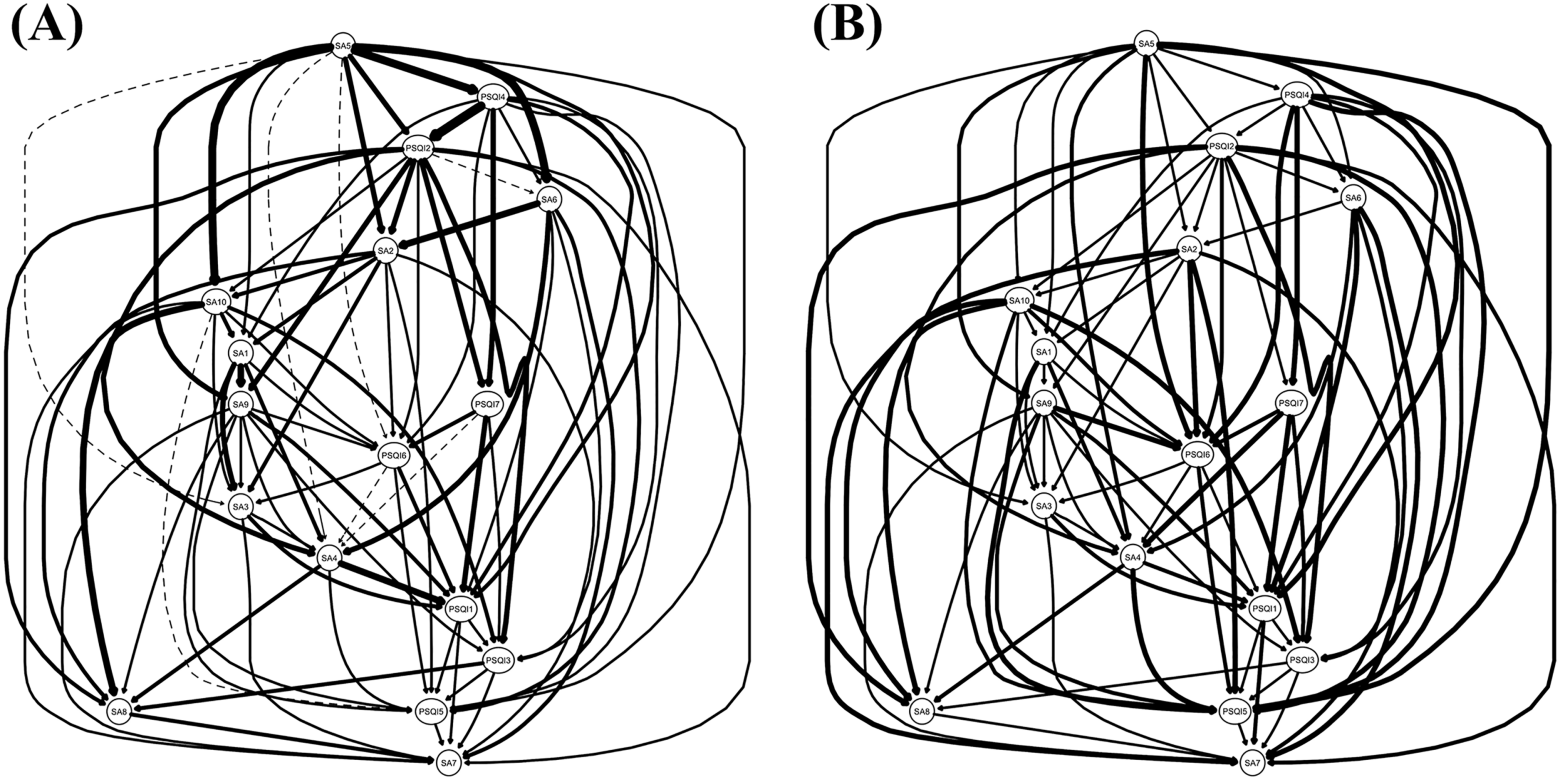
Directed Acyclic Graph (DAG) Networks for Smartphone Addiction and Sleep Disorder Symptoms. **(A)** DAG based on Bayesian Information Criterion (BIC). **(B)** DAG based on edge occurrence probability across 2000 bootstrap samples.

Fig 5A presents a Directed Acyclic Graph (DAG) for smartphone addiction and sleep disorder symptoms based on BIC values. The arrow thickness in the figure represents the change in the overall Bayesian Information Criterion (BIC) when the edge is removed, with a thicker arrow indicating a greater contribution to model fit. Node SA5 is positioned at the top of the DAG network and points to a total of 12 nodes, while no nodes point to it. This position suggests that SA5 may function as an independent initial node within the DAG network, potentially influencing or maintaining other symptoms. Therefore, SA5 can be regarded as a critical target for early intervention. In contrast, node SA7 is located at the bottom of the DAG network layout and does not point to any other nodes. This position implies that SA7 is more likely to be influenced by other symptoms, rather than exerting a statistical directional influence on them. Furthermore, Fig 5B displays the probability-based DAG. The arrow thickness in the figure indicates the probability of the directed connections occurring in the bootstrap networks. Specifically, the thicker arrow represents the higher probability of the corresponding directional association. Consistent with Fig 5A, Fig 5B shows that SA5 occupies the top position in the DAG network layout. It points to a total of 8 nodes while no node points to it, further suggesting the possibility that SA5 serves as an initial node of the network and a crucial target for intervention. Similarly, node SA7 is again positioned at the bottom and not pointing to other nodes, reinforcing its interpretation as a symptom more likely influenced by, rather than directionally influencing, other symptoms.

### Network comparison across genders

**Fig 6:**
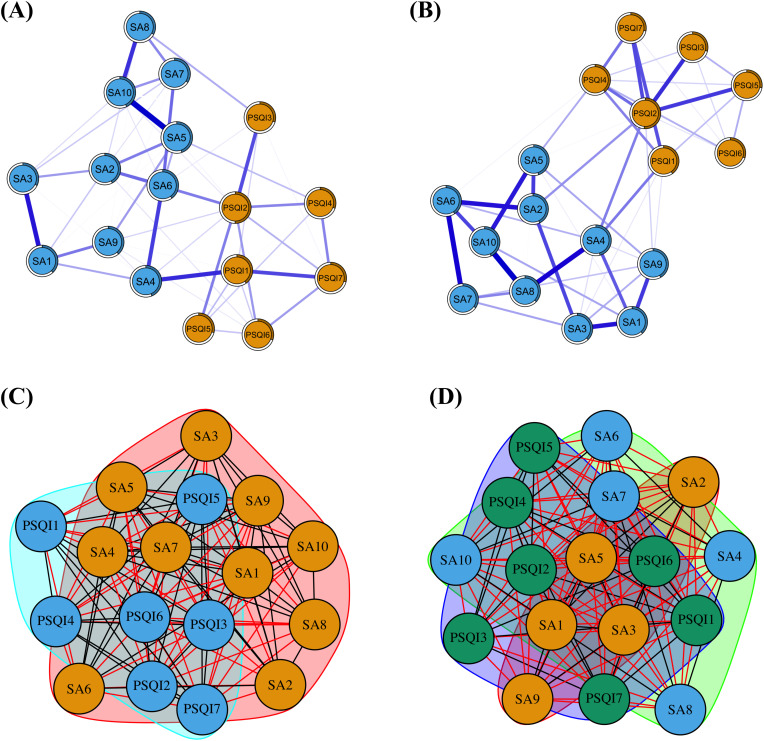
Symptom networks and community structures across genders. **(A)** Gaussian Graphical Model (GGM) network for males. **(B)** Gaussian Graphical Model (GGM) network for females. **(C)** Community structure for males. **(D)** Community structure for females.

Figs 6A and 6B illustrate the GGM networks of smartphone addiction and sleep disorder symptoms for male and female groups. Network comparison tests revealed no significant gender differences in global network structure, as both the network invariance test (M = 0.133, p = 0.112) and the global strength invariance test (M = 1.252, p = 0.243) were nonsignificant. Similarly, no significant gender differences were observed in the node centrality indices. However, the edge weight invariance test identified six edges that differed significantly by gender: SA1–SA2 (p = 0.047), SA4–SA6 (p = 0.037), SA4–SA8 (p = 0.003), SA5–SA10 (p = 0.045), SA7–PSQI3 (p = 0.041), and SA8–PSQI5 (p = 0.046). The specific results of the local invariance test are presented in S2 and S3 Tables. Furthermore, As shown in Figs 6C and 6D, community detection further revealed distinct clustering patterns between genders: symptoms in the male sample aggregated into two communities, whereas the female sample formed three. These results suggest that while the symptom networks are highly similar in global connectivity and nodal influence, local edge weights and symptom clustering patterns show potential gender differences.

## Discussion

GGM network analysis identified PSQI2 (sleep latency), SA2 (difficulty concentrating), and SA5 (impatience without phone) as core symptoms in the smartphone addiction and sleep disorders (SA-SD) comorbidity network. This suggests that these symptoms may directly influence other symptoms within the SA-SD network. These findings are consistent with prior research. For example, one study of Chinese adolescents with depression reported that sleep latency is recognized as a core symptom in the problematic smartphone use (PSU) and poor sleep quality (PSQ) comorbidity network [58]. Similarly, a study of 288 breast cancer patients found sleep latency as a core symptom within a network of sleep quality, anxiety, and depression [59]. However, not all studies support this conclusion. For instance, a cross-sectional survey of art students at a Chinese public university revealed uncontrollable worry, low energy, and excessive worry—rather than sleep latency—as the core symptoms within a comorbid network of anxiety, depression, sleep problems, and smartphone addiction [60]. Thus, while the present study has preliminarily identified core symptoms in the SA-SD comorbidity network, the specific core symptoms may vary across populations and cultural contexts. Notably, interpreting core symptoms as direct intervention targets requires caution. Although highly central nodes are often considered potential intervention targets in network analysis, centrality metrics primarily reflect the connectivity strength of nodes in cross-sectional networks and do not necessarily represent causal relationships or therapeutic efficacy [61,62]. Consequently, identifying core symptoms does not guarantee that they are the most effective or efficient intervention targets. Future studies should integrate longitudinal designs or experimental interventions to examine whether targeting these symptoms can indeed induce changes in the network structure and alleviate the comorbidity.

PSQI2 (sleep latency), PSQI1 (subjective sleep quality), and SA9 (longer use than intended) function as critical bridge symptoms linking smartphone addiction and sleep disorder symptoms. Among these, sleep latency plays a dual role as both a core and bridge symptom. It not only demonstrates high centrality within the SA-SD comorbidity network but also serves as a vital connector between the two symptom clusters. This finding is consistent with prior research. For instance, a network analysis of high school students confirmed that sleep latency exhibits high bridge strength in the comorbidity network of social media addiction and sleep quality [63]. Similarly, a study of depressed adolescents found that sleep latency was a bridge symptom in the network of non-suicidal self-injury and sleep quality [64]. However, not all studies support this conclusion. Other evidence suggests that symptoms such as daytime dysfunction and sleep disturbance may also serve as bridge symptoms connecting problematic smartphone use and poor sleep quality [58].

This study reveals the potential directional associations between sleep disorder and smartphone addiction symptoms. Based on directed acyclic graphs (DAG) analysis, most sleep disorder nodes point to smartphone addiction nodes, suggesting a directional association between sleep disorders and smartphone addiction. This result is highly consistent with findings from existing longitudinal studies. For example, a longitudinal study of adolescents using the cross-lagged panel model (CLPM) found that sleep problems at time point T1 significantly predicted problematic smartphone use at time point T2 [20]. Similarly, another longitudinal study of students also showed that higher baseline scores on the Insomnia Severity Index (ISI) predicted more severe mobile phone addiction one year later [65]. Taken together, these findings suggest that improving sleep quality may help alleviate smartphone addiction. Therefore, interventions targeting SA-SD comorbidity should prioritize the improvement and optimization of sleep quality. It should be noted, however, that the DAG analysis in this study is based on cross-sectional data; thus, inferred directional associations should be considered preliminary, and future research needs to rely on longitudinal design to further verify these relationships.

The Directed Acyclic Graph (DAG) model found that SA5 (impatience without phone) is an initial node in the Bayesian network for smartphone addiction and sleep disorders. According to the Compensatory Internet Use Theory (CIUT), individuals frequently utilize smartphones to regulate negative emotions or to escape real-life pressures [29]. SA5 (impatience without phone) accurately reflects the anxiety and fear related to losing this immediate coping mechanism, which in turn sustains anticipatory anxiety and drives addictive behavior. Consequently, SA5 (impatience without phone) may influence subsequent symptoms such as SA2 (difficulty concentrating), SA10 (others’ comments on overuse), PSQI4 (habitual sleep efficiency), and PSQI2 (sleep latency). Therefore, in future longitudinal or intervention studies, priority should be given to SA5 to explore whether it can affect the development of other symptoms at the source. However, it should be emphasized that the above directional associations are based on statistical inference of cross-sectional data and cannot yet be equated with causal relationships.

The ability of Bayesian networks to infer directional associations from cross-sectional data requires further empirical validation due to two key limitations. First, Bayesian networks rely on certain assumptions [66] that are often difficult to satisfy in the context of complex mental disorders [67]. Second, directional inferences derived from Bayesian networks—represented here as directed acyclic graphs (DAG)—can only estimate unidirectional relationships between symptoms and do not account for potential feedback loops. Given that mental disorder symptoms frequently exhibit mutually influencing bidirectional relationships, Bayesian networks may fail to capture direct or robust directional associations [68]. In summary, directional associations derived from cross-sectional data should be considered as exploratory hypotheses rather than exact causal conclusions. Future research should employ longitudinal design or experimental interventions to further validate the directional relationship proposed in this study.

Although previous studies have shown differences in smartphone use and sleep disorders between males and females [56], the network comparison test in this study showed no significant gender differences in the comorbidity network structure of smartphone addiction and sleep disorders among college students. This finding is consistent with previous research results. For example, a network comparison test among college art students focusing on anxiety, depression, sleep problems, and smartphone addiction found no significant gender differences in network structure [60]. However, community detection revealed that the male comorbidity network was divided into two communities, while the female network was divided into three communities. This finding indicates a significant gender difference in the clustering structure of symptoms. This difference in community structure provides an important basis for developing gender specific prevention and intervention strategies. Specifically, male college students exhibit a higher degree of symptom integration, and thus intervention strategies can adopt a more integrated and holistic approach to behavior management. In contrast, female college students show more pronounced symptom differentiation, so intervention strategies may need to be more modular and targeted.

Finally, this study has several strengths. First, it comprehensively employs the Gaussian Graphical Model (GGM) and the Directed Acyclic Graph (DAG) to systematically analyze the comorbidity network of smartphone addiction and sleep disorder symptoms among Chinese college students. This approach not only illustrates the cross-sectional associations among symptoms but also provides a foundation for inferring potential directional associations between them. Second, this study focuses specifically on Chinese college students—a population at high risk for smartphone addiction [69]. By identifying core symptoms, bridge symptoms, and initial driving nodes within the comorbid network, it provides an empirical basis for developing targeted intervention strategies for this population. This study also has several limitations. First, it employs cross-sectional data for analysis. While this approach can reveal correlations between symptoms, it does not ascertain the causal relationships among them. Consequently, future research should utilize longitudinal panel data to construct temporal or cross-lagged networks, thereby elucidating the causal mechanisms underlying the mutual influence of symptoms. Second, convenience sampling was used in this study, which may limit the conclusions to specific samples. Therefore, future research should validate these findings in a wider and more representative sample. Furthermore, this study relied on self-report scales (SAS-SV and PSQI) to collect data. Self-report data are susceptible to recall bias and social desirability bias, and data from a single source are subject to common method bias. Therefore, future research should employ more objective measurement tools, such as smartphone usage logs, wearable sleep monitoring devices, and multimodal behavioral and physiological data, to more accurately reveal the true associations among symptoms.

## Conclusion

Our research findings revealed that symptoms of smartphone addiction and sleep disorder are highly prevalent in this population. Based on Gaussian Graphical Model (GGM) network analysis, the comorbidity network of smartphone addiction and sleep disorders exhibited high overall connection density and global strength. Within this network, PSQI2 (sleep latency), SA2 (difficulty concentrating), and SA5 (impatience without phone) were identified as core symptoms, while PSQI2 (sleep latency), PSQI1 (subjective sleep quality), and SA9 (longer use than intended) were identified as bridge symptoms. Additionally, Directed Acyclic Graph (DAG) analysis suggested a directional correlation between sleep disorders and smartphone addiction. Network comparison analysis revealed no significant gender differences in the GGM comorbidity structure between male and female university students. However, differences were observed in their internal community divisions, highlighting the importance of considering gender factors in intervention strategies.

The findings of this study have important clinical implications. First, the network analysis identified key core and bridge symptoms, providing precise targets for developing tailored intervention strategies. Intervening in core and bridge symptoms is expected to significantly improve the overall sleep quality and mental health of college students. Second, the Directed Acyclic Graph (DAG) results offer a statistical directionality, suggesting a potential priority for prevention strategies. This study found that sleep disorder symptoms are more likely to point to smartphone addiction symptoms. Therefore, the primary goal of prevention should be to detect and improve sleep problems. Finally, the research findings can inform government agencies in developing more effective mental health policies, thereby promoting the overall well-being of college students and providing scientific decision-making support for building healthy campuses.

## Supporting information

S1 Table Stability of Centrality Indices. This table summarizes the stability of network centrality indices using the case-dropping subset bootstrap procedure. For each subsample level, the table reports the number of remaining persons (nPerson), the corresponding percentage of the full sample dropped (Drop%), and the number of bootstrap samples drawn (n).
(DOCX)

S2 Table Results of Centrality Invariance Tests for Expected Influence. This table displays the invariance test results of the expected impact centrality measure for each node in the resampled sample.
(DOCX)

S3 Table Results of Edge Weight Invariance Tests. This table presents the complete results of the invariance tests for all estimated edge weights between symptoms across the compared groups. The p-value for each edge indicates the statistical significance of its weight difference between groups. Edges marked with an asterisk (*) are those with a significant difference at p < 0.05.
(DOCX)
