# Supplementary material for: Network analysis of smartphone addiction and sleep disorder symptoms in Chinese college students
Source: PLoS One. 2026 May 22;21(5):e0349016. doi: 10.1371/journal.pone.0349016 (PMC13196963; doi:10.1371/journal.pone.0349016)
Supplement: S1 Table — This table summarizes the stability of network centrality indices using the case-dropping subset bootstrap procedure. For each subsample level, the table reports the number of remaining persons (nPerson), the corresponding percentage of the full sample dropped (Drop%), and the number of bootstrap samples drawn (n). (DOCX) [file pone.0349016.s001.docx]

****Table S1: Stability of Centrality Indices****

| **Subset** | **Sample Size (nPerson)** | **Percentage Dropped (Drop%)** | **of Subsets (n)** |
| --- | --- | --- | --- |
| 1 | 460 | 75.0% | 172 |
| 2 | 604 | 67.2% | 162 |
| 3 | 747 | 59.4% | 147 |
| 4 | 890 | 51.7% | 138 |
| 5 | 1034 | 43.9% | 130 |
| 6 | 1177 | 36.1% | 146 |
| 7 | 1320 | 28.3% | 145 |
| 8 | 1463 | 20.6% | 152 |
| 9 | 1607 | 12.8% | 149 |
| 10 | 1750 | 5.0% | 159 |

***Note:*** *This table summarizes the stability of network centrality indices using the case-dropping subset bootstrap procedure. For each subsample level, the table reports the number of remaining persons (nPerson), the corresponding percentage of the full sample dropped (Drop%), and the number of bootstrap samples drawn (n).*
