# Supplementary material for: Network analysis of smartphone addiction and sleep disorder symptoms in Chinese college students
Source: PLoS One. 2026 May 22;21(5):e0349016. doi: 10.1371/journal.pone.0349016 (PMC13196963; doi:10.1371/journal.pone.0349016)
Supplement: S2 Table — This table displays the invariance test results of the expected impact centrality measure for each node in the resampled sample. (DOCX) [file pone.0349016.s002.docx]

**Table S2. Results of Centrality Invariance Tests for Expected Influence**

| **Node** | **Test Statistic (C)** | **p-value** |
| --- | --- | --- |
| SA1 | -0.0415 | 0.582 |
| SA2 | 0.1114 | 0.183 |
| SA3 | -0.0073 | 0.907 |
| SA4 | 0.0116 | 0.851 |
| SA5 | -0.0296 | 0.691 |
| SA6 | 0.0099 | 0.916 |
| SA7 | -0.0452 | 0.563 |
| SA8 | -0.0376 | 0.642 |
| SA9 | 0.0650 | 0.407 |
| SA10 | -0.0508 | 0.504 |
| PSQI1 | 0.0458 | 0.578 |
| PSQI2 | 0.0077 | 0.923 |
| PSQI3 | -0.0651 | 0.409 |
| PSQI4 | -0.1467 | 0.069 |
| PSQI5 | -0.0214 | 0.745 |
| PSQI6 | 0.0093 | 0.886 |
| PSQI7 | 0.1049 | 0.126 |

***Note:*** *This table displays the invariance test results of the expected impact centrality measure for each node in the resampled sample.*
