# Supplementary material for: Network analysis of smartphone addiction and sleep disorder symptoms in Chinese college students
Source: PLoS One. 2026 May 22;21(5):e0349016. doi: 10.1371/journal.pone.0349016 (PMC13196963; doi:10.1371/journal.pone.0349016)
Supplement: S3 Table — This table presents the complete results of the invariance tests for all estimated edge weights between symptoms across the compared groups. The p-value for each edge indicates the statistical significance of its weight difference between groups. Edges marked with an asterisk (*) are those with a significant difference at p < 0.05. (DOCX) [file pone.0349016.s003.docx]

**Table S3: Results of Edge Weight Invariance Tests**

|  | **Node1** | **Node2** | **p-value** | **Test statistic E** |
| --- | --- | --- | --- | --- |
| **1** | SA1 | SA2 | 0.047* | 0.080 |
| **2** | SA1 | SA3 | 0.322 | 0.040 |
| **3** | SA2 | SA3 | 0.529 | 0.028 |
| **4** | SA1 | SA4 | 0.562 | 0.025 |
| **5** | SA2 | SA4 | 0.121 | 0.016 |
| **6** | SA3 | SA4 | 0.785 | 0.011 |
| **7** | SA1 | SA5 | 0.911 | 0.005 |
| **8** | SA2 | SA5 | 0.758 | 0.012 |
| **9** | SA3 | SA5 | 0.960 | 0.003 |
| **10** | SA4 | SA5 | 0.599 | 0.019 |
| **11** | SA1 | SA6 | 1.000 | 0.000 |
| **12** | SA2 | SA6 | 0.616 | 0.022 |
| **13** | SA3 | SA6 | 0.994 | 0.000 |
| **14** | SA4 | SA6 | 0.037* | 0.092 |
| **15** | SA5 | SA6 | 0.051 | 0.083 |
| **16** | SA1 | SA7 | 0.328 | 0.016 |
| 17 | SA2 | SA7 | 0.420 | 0.034 |
| 18 | SA3 | SA7 | 0.830 | 0.010 |
| 19 | SA4 | SA7 | 0.664 | 0.018 |
| 20 | SA5 | SA7 | 0.447 | 0.030 |
| 21 | SA6 | SA7 | 0.403 | 0.034 |
| 22 | SA1 | SA8 | 1.000 | 0.000 |
| 23 | SA2 | SA8 | 0.386 | 0.036 |
| 24 | SA3 | SA8 | 0.254 | 0.016 |
| 25 | SA4 | SA8 | 0.003* | 0.133 |
| 26 | SA5 | SA8 | 0.079 | 0.071 |
| 27 | SA6 | SA8 | 0.723 | 0.015 |
| 28 | SA7 | SA8 | 0.701 | 0.018 |
| 29 | SA1 | SA9 | 0.791 | 0.011 |
| 30 | SA2 | SA9 | 0.261 | 0.035 |
| 31 | SA3 | SA9 | 0.166 | 0.059 |
| 32 | SA4 | SA9 | 0.988 | 0.001 |
| 33 | SA5 | SA9 | 0.605 | 0.022 |
| 34 | SA6 | SA9 | 0.234 | 0.014 |
| 35 | SA7 | SA9 | 0.535 | 0.026 |
| 36 | SA8 | SA9 | 0.375 | 0.038 |
| 37 | SA1 | SA10 | 0.302 | 0.043 |
| 38 | SA2 | SA10 | 0.302 | 0.042 |
| 39 | SA3 | SA10 | 0.283 | 0.048 |
| 40 | SA4 | SA10 | 1.000 | 0.000 |
| 41 | SA5 | SA10 | 0.045* | 0.081 |
| 42 | SA6 | SA10 | 0.065 | 0.076 |
| 43 | SA7 | SA10 | 0.076 | 0.071 |
| 44 | SA8 | SA10 | 0.867 | 0.007 |
| 45 | SA9 | SA10 | 0.806 | 0.011 |
| 46 | SA1 | PSQI1 | 0.815 | 0.010 |
| 47 | SA2 | PSQI1 | 1.000 | 0.000 |
| 48 | SA3 | PSQI1 | 0.545 | 0.025 |
| 49 | SA4 | PSQI1 | 0.078 | 0.079 |
| 50 | SA5 | PSQI1 | 0.126 | 0.083 |
| 51 | SA6 | PSQI1 | 0.198 | 0.047 |
| 52 | SA7 | PSQI1 | 0.391 | 0.038 |
| 53 | SA8 | PSQI1 | 1.000 | 0.000 |
| 54 | SA9 | PSQI1 | 0.822 | 0.010 |
| 55 | SA10 | PSQI1 | 1.000 | 0.000 |
| 56 | SA1 | PSQI2 | 1.000 | 0.000 |
| 57 | SA2 | PSQI2 | 0.415 | 0.032 |
| 58 | SA3 | PSQI2 | 1.000 | 0.000 |
| 59 | SA4 | PSQI2 | 0.357 | 0.036 |
| 60 | SA5 | PSQI2 | 0.910 | 0.003 |
| 61 | SA6 | PSQI2 | 0.099 | 0.057 |
| 62 | SA7 | PSQI2 | 0.430 | 0.044 |
| 63 | SA8 | PSQI2 | 0.178 | 0.038 |
| 64 | SA9 | PSQI2 | 0.901 | 0.005 |
| 65 | SA10 | PSQI2 | 0.125 | 0.073 |
| 66 | PSQI1 | PSQI2 | 0.209 | 0.053 |
| 67 | SA1 | PSQI3 | 0.053 | 0.021 |
| 68 | SA2 | PSQI3 | 1.000 | 0.000 |
| 69 | SA3 | PSQI3 | 1.000 | 0.000 |
| 70 | SA4 | PSQI3 | 1.000 | 0.000 |
| 71 | SA5 | PSQI3 | 1.000 | 0.000 |
| 72 | SA6 | PSQI3 | 0.054 | 0.083 |
| 73 | SA7 | PSQI3 | 0.041* | 0.080 |
| 74 | SA8 | PSQI3 | 0.143 | 0.074 |
| 75 | SA9 | PSQI3 | 0.382 | 0.036 |
| 76 | SA10 | PSQI3 | 0.191 | 0.079 |
| 77 | PSQI1 | PSQI3 | 0.535 | 0.027 |
| 78 | PSQI2 | PSQI3 | 0.772 | 0.013 |
| 79 | SA1 | PSQI4 | 0.771 | 0.013 |
| 80 | SA2 | PSQI4 | 1.000 | 0.000 |
| 81 | SA3 | PSQI4 | 1.000 | 0.000 |
| 82 | SA4 | PSQI4 | 1.000 | 0.000 |
| 83 | SA5 | PSQI4 | 0.946 | 0.003 |
| 84 | SA6 | PSQI4 | 0.557 | 0.024 |
| 85 | SA7 | PSQI4 | 1.000 | 0.000 |
| 86 | SA8 | PSQI4 | 1.000 | 0.000 |
| 87 | SA9 | PSQI4 | 1.000 | 0.000 |
| 88 | SA10 | PSQI4 | 1.000 | 0.000 |
| 89 | PSQI1 | PSQI4 | 0.171 | 0.058 |
| 90 | PSQI2 | PSQI4 | 0.877 | 0.007 |
| 91 | PSQI3 | PSQI4 | 0.319 | 0.046 |
| 92 | SA1 | PSQI5 | 0.079 | 0.074 |
| 93 | SA2 | PSQI5 | 0.837 | 0.008 |
| 94 | SA3 | PSQI5 | 0.407 | 0.000 |
| 95 | SA4 | PSQI5 | 0.061 | 0.053 |
| 96 | SA5 | PSQI5 | 0.234 | 0.016 |
| 97 | SA6 | PSQI5 | 1.000 | 0.000 |
| 98 | SA7 | PSQI5 | 0.630 | 0.012 |
| 99 | SA8 | PSQI5 | 0.046* | 0.015 |
| 100 | SA9 | PSQI5 | 0.345 | 0.043 |
| 101 | SA10 | PSQI5 | 0.089 | 0.055 |
| 102 | PSQI1 | PSQI5 | 0.819 | 0.010 |
| 103 | PSQI2 | PSQI5 | 0.737 | 0.014 |
| 104 | PSQI3 | PSQI5 | 0.386 | 0.038 |
| 105 | PSQI4 | PSQI5 | 0.763 | 0.013 |
| 106 | SA1 | PSQI6 | 0.633 | 0.023 |
| 107 | SA2 | PSQI6 | 0.299 | 0.043 |
| 108 | SA3 | PSQI6 | 0.365 | 0.022 |
| 109 | SA4 | PSQI6 | 0.195 | 0.023 |
| 110 | SA5 | PSQI6 | 0.165 | 0.046 |
| 111 | SA6 | PSQI6 | 0.382 | 0.017 |
| 112 | SA7 | PSQI6 | 0.216 | 0.028 |
| 113 | SA8 | PSQI6 | 1.000 | 0.000 |
| 114 | SA9 | PSQI6 | 0.600 | 0.023 |
| 115 | SA10 | PSQI6 | 0.247 | 0.031 |
| 116 | PSQI1 | PSQI6 | 0.416 | 0.035 |
| 117 | PSQI2 | PSQI6 | 0.220 | 0.052 |
| 118 | PSQI3 | PSQI6 | 0.208 | 0.056 |
| 119 | PSQI4 | PSQI6 | 0.388 | 0.040 |
| 120 | PSQI5 | PSQI6 | 0.989 | 0.000 |
| 121 | SA1 | PSQI7 | 1.000 | 0.000 |
| 122 | SA2 | PSQI7 | 1.000 | 0.000 |
| 123 | SA3 | PSQI7 | 1.000 | 0.000 |
| 124 | SA4 | PSQI7 | 0.258 | 0.036 |
| 125 | SA5 | PSQI7 | 0.281 | 0.026 |
| 126 | SA6 | PSQI7 | 1.000 | 0.000 |
| 127 | SA7 | PSQI7 | 1.000 | 0.000 |
| 128 | SA8 | PSQI7 | 1.000 | 0.000 |
| 129 | SA9 | PSQI7 | 0.282 | 0.015 |
| 130 | SA10 | PSQI7 | 0.941 | 0.002 |
| 131 | PSQI1 | PSQI7 | 0.418 | 0.037 |
| 132 | PSQI2 | PSQI7 | 0.316 | 0.042 |
| 133 | PSQI3 | PSQI7 | 0.613 | 0.022 |
| 134 | PSQI4 | PSQI7 | 0.782 | 0.011 |
| 135 | PSQI5 | PSQI7 | 0.445 | 0.021 |
| 136 | PSQI6 | PSQI7 | 0.277 | 0.053 |

***Note:*** *This table presents the complete results of the invariance tests for all estimated edge weights between symptoms across the compared groups. The p-value for each edge indicates the statistical significance of its weight difference between groups. Edges marked with an asterisk (*) are those with a significant difference at p < 0.05.*
